# Antagonistic Action of Lactobacilli and Bifidobacteria in Relation to *Staphylococcus aureus* and Their Influence on the Immune Response in Cases of Intravaginal Staphylococcosis in Mice

**DOI:** 10.1007/s12602-012-9093-z

**Published:** 2012-02-25

**Authors:** Liudmyla Lazarenko, Lidiia Babenko, Liubov Shynkarenko Sichel, Valentyn Pidgorskyi, Viktoriia Mokrozub, Olga Voronkova, Mykola Spivak

**Affiliations:** 1 D.K. Zabolotny Institute of Microbiology and Virology of the National Academy of Sciences of Ukraine, Kyiv, 02154 Ukraine; 2 O. Honchar Dnipropetrovsk National University, Dnipropetrovsk, 49000 Ukraine; 3 Pure Research Products, LLC, Boulder, CO USA

**Keywords:** *Lactobacillus*, *Bifidobacterium*, *Staphylococcus*, Vagina, Mouse, Immunity

## Abstract

The antibacterial activity of *Lactobacillus casei* IMV B-7280, *Lact. acidophilus* IMV B-7279, *Bifidobacterium longum* VK1, and *B. bifidum* VK2 strains or their various compositions in relation to *Staphylococcus aureus* in vitro and on models of experimental intravaginal staphylococcosis of mice was determined. It was found that under the influence of these strains and their various compositions, the in vitro growth of *Staph. aureus* was inhibited, and the number of colonies of *Staph. aureus* plated from the vagina of infected mice was significantly reduced. The antibacterial activity of these strains separately and in compositions correlated with their ability to improve the performance of the immune response. These strains were the most effective in the following compositions: *Lact. casei* IMV B-7280—*B. longum* VK1—*B. bifidum* VK2. Strains of *Lact. casei* IMV B-7280, *Lact. acidophilus* IMV B-7279, *B. bifidum* VK2, and *B. longum* VK1 are prospective components of future probiotic drugs efficient in treating staphylococcosis and for immunity correction.

## Introduction

Dysfunction of the immune system that emerges as the result of changes in microbial ecology, widespread use of modern chemopreparations of various natures, disruption of the normal microbiota, etc., is one of the major causes of increasingly hostile opportunistic commensal pathogens, with subsequent development of infectious diseases of the urogenital tract, including the anogenital area. The normal biota of the vagina mainly consists of lactobacilli and a smaller number of bifidobacteria, staphylococci, streptococci, coryneforms, enterococci, enterobacteria, etc. [19]. The vaginal microflora is now more frequently viewed as an “ecosystem” [18]. The vaginal lactic acid bacteria colonize the mucous membranes, maintain the proper acidity (pH 4.3–4.7), control pathogens that cause urinary tract infections and/or sexually transmitted diseases and may also affect the development of the immune response to causative agents of infectious diseases [18, 19]. Therefore, disruption of the normal vaginal microflora, especially due to reduction in the number or activity of lactobacilli [30], frequently causes activation of aggressive forms of opportunistic commensal pathogens, resulting in the development of vaginosis or uncomplicated urinary tract infections, as well as the emergence of other pathological conditions.

It is known that uncomplicated infections of urinary tract and vaginosis are often caused by opportunistic commensal bacteria of the *Staphylococcus* genus [5, 15]. Staphylococcosis usually develops in people with reduced nonspecific immunological resistance, as well as in people who received large doses of immune suppressants, antibiotics, hormones, X-rays, etc. The latter has led to emergence of resistant staphylococci. Frequent regressive uncomplicated urinary tract infection can cause serious diseases, such as nephritis, kidney damage, etc. Long-lasting bacterial vaginosis caused by staphylococci is associated with a high risk of development of sexually transmitted infectious diseases, which may increase the risk of late miscarriage [7, 14, 26].

Therefore, developing alternative nature-derived treatment(s) for patients with uncomplicated urinary tract infections and vaginosis is of the utmost concern. This treatment may include healthy vaginal lactic acid bacteria with expressed antibacterial and immune modulatory properties. There are only a few known strains of lactobacilli that demonstrated a therapeutic effect in cases of urogenital infectious diseases on experimental models and in patients’ treatment [8].

We have previously characterized the following strains of lactobacilli and bifidobacteria: *Lactobacillus casei* IMV B-7280, *Lact.* *acidophilus* IMV B-7279, *Bifidobacterium longum* VK1, and *B. bifidum* VK2. It was found that these strains had in vitro antagonistic effects in relation to a wide range of pathogenic and opportunistic microorganisms, including causative agents of infectious diseases of the urogenital tract. Furthermore, on the model of intact mice, it was shown that in vivo they effectively induced production of endogenous interferon and activated cells of the phagocytic system, without affecting the production of the pro-inflammatory cytokine tumor necrosis factor-α [27].

The aim of this study was to investigate the anti-staphylococcal activity of the *Lact. casei* IMV B-7280, *Lact. acidophilus* IMV B-7279, *B. longum* VK1 and *B. bifidum* VK2 strains and their compositions on the model of experimental intravaginal staphylococcosis of mice, and determine their influence on innate immunity indicators.

## Materials and Methods

### Strains

*Lact. casei* IMV B-7280, *Lact. acidophilus* IMV B-7279, *B. longum* VK1 and *B. bifidum* VK2 strains were used both individually and in various compositions. These strains were previously selected by us from associated cultures in the course of laboratory study of fermented biological materials. The study was performed using bacteria lyophilized in Cuddon Freeze Dryer FD1500 (New Zealand). Before each experiment, the viability of the probiotic cultures was tested by monitoring their growth on the Man-Rogosa-Sharpe (MRS) agar medium at 37 °C for 24–48 h.

*Staph. aureus* 8325-4 (kindly provided to us by Professor V. S. Zuyeva, N. F. Gamaleya Institute of Epidemiology and Microbiology, Russian Federation) had plasmid-based resistance to gentamicin, allowing it to be separated from other strains of vaginal staphylococcus obtained from the environment through the use of selective media containing this antibiotic.

*Staph. aureus* 8325-4 was grown on selective medium for staphylococci (BAIRD-PARKER-Agar, Merck, Germany) containing gentamicin (15 μg/ml) at 37 °C for 24 h.

### In Vitro Antagonistic Activity Assays

The antagonistic activity of *Lact. casei* IMV B**-**7280, *Lact.* *acidophilus* IMV B-7279, *B. longum* VK1 and *B. bifidum* VK2 strains was determined in vitro in relation to the laboratory collection strains *Staph. aureus* 209-P, *Staph. aureus* 43 and *Staph. aureus* 8325-4 (D. K. Zabolotny Institute of Microbiology and Virology of the National Academy of Sciences of Ukraine).

In the study of antagonistic activity of probiotic cultures, the method of perpendicular strokes on the MRS medium [20] was used. Test cultures of lactobacillus or bifidobacteria strains were collected after 24 h of cultivation. The degree of sensitivity of the test cultures was evaluated according to the size of the zones of growth inhibition: 5–15 mm—low-sensitive, 15–20 mm—moderately sensitive, 30–40 mm—highly sensitive.

### Model of Staphylococcal Intravaginal Infection and Treatment of Mice with Lactobacilli and/or Bifidobacteria

Experimental studies were performed on six-week-old female BALB/c mice, synchronized in their estral cycle. All studies were performed taking into account the rules of the European Convention for the protection of vertebrate animals. Staphylococcosis was modeled through intravaginal administration of the *Staph. aureus* 8325-4 daily culture to mice, in doses of 5 × 10^7^ cells per animal. The following clinical manifestations of the infection process were observed in the infected mice: significant increase in whitish mucous secretions of the vagina, elevation of body temperature, inactivity, and loss of appetite.

Twenty-four hours after infection, mice were given an intravaginal injection of a suspension of lyophilized lactobacillus and/or bifidobacteria cells in saline solution at a dose of 1 × 10^6^ cells per animal, once per day for 7 days. Strains were injected individually and in the following combinations: *Lact. casei* IMV B-7280—*Lact.* *acidophilus* IMV B-7279; *Lact. casei* IMV B-7280—*B. longum* VK1; *Lact. casei* IMV B-7280—*B. bifidum* VK2; *Lact.* *acidophilus* IMV B-7279—*B. longum* VK1; *Lact.* *acidophilus* IMV B-7279—*B. bifidum* VK2; *Lact. casei* IMV B-7280—*B. longum* VK1—*B. bifidum* VK2; *Lact. casei* IMV B-7280—*B. longum* VK1—*Lact. acidophilus* IMV B-7279; *Lact. acidophilus*—*B. longum* VK1—*B. bifidum* VK2; *Lact. casei* IMV B-7280—*B. bifidum* VK2—*Lact. acidophilus* IMV B-7279; *Lact. casei* IMV B-7280—*B. bifidum* VK2—*B. longum* VK1—*Lact. acidophilus* IMV B-7279 in equal proportion. A separate group was formed by the infected mice that did not receive these strains or their combinations, but received the saline intravaginally. The control group included intact mice.

### Quantification of the Antibacterial Activity In Vivo

On the first, third, sixth, ninth and twelfth day after the injection of the lactobacillus and/or bifidobacteria strains, alone or in various combinations, into the mice, *Staph. aureus* 8325-4 was collected from the vagina and plated onto a selective medium for staphylococci containing gentamicin. The material was collected using standardized sterile cotton tampons. Swabs from each tampon were performed with 1 ml of saline. After cultivation at 37 °C for 24 h, the number of colony forming units was counted, given that one such colony corresponds to one bacterium.

### Determining the Number of T- and B-Lymphocytes in the Spleen

On the first, third, sixth and ninth day after the injection of the lactobacillus and/or bifidobacteria strains, alone or in combinations, into the mice, the spleens were extracted from the killed mice, and suspensions of splenocytes were prepared in RPMI-1640 culture medium. Leukocytes were extracted from the spleen cell suspension by fractionating cells in ficoll-verohrafin density gradient (ρ = 1.077 g/cm^3^) by centrifuging (on the centrifuge/vortex Multi-Spin MSC-3000) at 400*g* for 15 min. The cells were then washed twice in the RPMI-1640 culture medium by centrifuging at 400*g* for 10 min. Surface antigens of T- and B-lymphocytes were investigated with the help of the direct immunofluorescence method. Monoclonal antibodies to CD3+, CD4+, CD8+ and CD19+ antigens (MACS, Miltenyi Biotec, Germany) were used in the work. Calculation of T- and B-lymphocytes and analysis of the results were performed on a FACStar Plus cytofluorometer (Becton–Dickinson, USA).

### Statistics

All digital data received were processed with the help of the Origin Pro 8.5. software through analysis of variance. Numerical data were represented as arithmetic average and standard error (M ± m). The null hypothesis for the control and experimental comparative groups was checked using Wilcoxon–Mann–Whitney (U) and Kolmogorov–Smirnov nonparametric criteria. The differences between the groups were considered statistically meaningful at *P* < 0.05.

## Results

### Antagonistic Action of Lactobacilli and Bifidobacteria in Relation to *Staph. aureus*

It was shown that all the tested probiotic cultures possess antagonistic activity in vitro in relation to the laboratory strains of *Staph. aureus*, including *Staph. aureus* 8325-4 (Table 1). Zones of growth inhibition of the laboratory test cultures varied for the *Lact.* *acidophilus* IMV B-7279 strain within 14–35 mm, for *Lact. casei* IMV B-7280 within 11–42 mm, for *B. bifidum* VK-1 within 17–39 mm, and for *B. longum* VK-2 within 5–20 mm. The growth of some test cultures was inhibited almost completely. Thus, *Lact.* *acidophilus* IMV B-7279 almost completely prevented the growth of *Staph. aureus* 8325-4 and other *Staph. aureus* spp., while *Lact. casei* IMV B-7280 prevented the growth of *Staph. aureus* spp. *B. bifidum* VK-1 also caused relatively large zones of growth inhibition when tested against *Staph. aureus* *43* and other *Staph. aureus* spp*.*

**Table 1:** Antagonistic activity of *Lact. casei* IMV B-7280, *Lact.* *acidophilus* IMV B-7279, *B. longum* VK1, and *B. bifidum* VK2 in relation to *S. aureus* in vitro

Test-culture	Area of growth retardation, mm			
	*Lact. acidophilus* IMV B-7279	*Lact. casei* IMV B-7280	*B. bifidum* VK-1	*B. longum* VK-2
*Staph. aureus* 209-P	22.0 ± 1.3	16.0 ± 0.9	17.0 ± 1.1	16.0 ± 1.0
*Staph. aureus* 43	14.0 ± 2.1	21.0 ± 1.7	31.0 ± 1.2	5.0 ± 0.8
*Staph. aureus* 8325-4	36.0 ± 1.9	11.0 ± 0.8	24.0 ± 2.1	11.0 ± 2.4
*Staph. aureus* spp*.*	34.0 ± 2.0	42.0 ± 1.3	39.0 ± 1.5	20.0 ± 1.2

*Lact.* *acidophilus* IMV B-7279 and *B. bifidum* VK-1 had the most effective antagonistic action in relation to *Staph. aureus* 8325-4 in vitro, while *Lact. casei* IMV B-7280 and *B. longum* VK-2 were the least effective. Thus, *S. aureus* 8325-4 was highly sensitive to *Lact.* *acidophilus* IMV B-7279, moderately sensitive to *B. bifidum* VK-1 and low-sensitive to *Lact. casei* IMV B-7280 and *B. longum* VK-2.

**Fig. 1:**
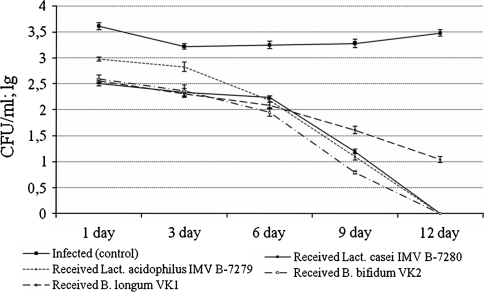
Number of colonies of *Staph. aureus* 8325-4, which was sowed from the vagina of the infected mice after receiving intravaginal injection of probiotic strains of lactobacilli and bifidobacteria, each of them separately

The following data were obtained from the study of the anti-staphylococcal activity of the probiotic strains in vivo. It was found that *Staph. aureus* 8325-4 was recovered from the vagina of infected mice who did not receive probiotic cultures or their compositions during the entire period of observation (days 1–12) (Fig. 1). At the same time, after injection of probiotic cultures in monocultures or in various compositions into the infected mice, the number of colonies of *Staph. aureus* 8325-4 decreased significantly compared to the infected mice that did not receive these strains or their compositions. Thus, after injection of *Lact.* *acidophilus* IMV B-7279, *Lact. casei* IMV B-7280, *B. longum* VK1 or *B. bifidum* VK2 separately to mice, a decrease in the number of *Staph. aureus* 8325-4 colonies, which was recovered from the vagina, was observed from the first day and throughout the entire subsequent period of observation. However, if we compare these probiotic cultures on an individual basis (Fig. 1), on the first and third days the anti-staphylococcal activity of *Lact. casei* IMV B-7280, *B. longum* VK1 and *B. bifidum* VK2 was higher than of *Lact.* *acidophilus* IMV B-7279 (*P* < 0.05). On the sixth and ninth days *Staph. aureus* 8325-4 was recovered in the smallest amount from the vagina of the infected mice who received *B. bifidum* VK2 (*P* < 0.05). On the twelfth day, *Staph. aureus* 8325-4 was eliminated completely from the vagina after injection of *Lact. casei* IMV B-7280, *B. bifidum* VK2 or *Lact. acidophilus* IMV B-7279 into the infected mice, but was still recovered in small amounts from the vagina of the infected mice who received *B. longum* VK1. These data show that the efficiency of anti-staphylococcal actions of certain probiotic cultures in vivo can be assessed as follows: *B. bifidum* VK2 > *Lact. casei* IMV B-7280 > *B. longum* VK1/*Lact.* *acidophilus* IMV B-7279.

**Fig. 2:**
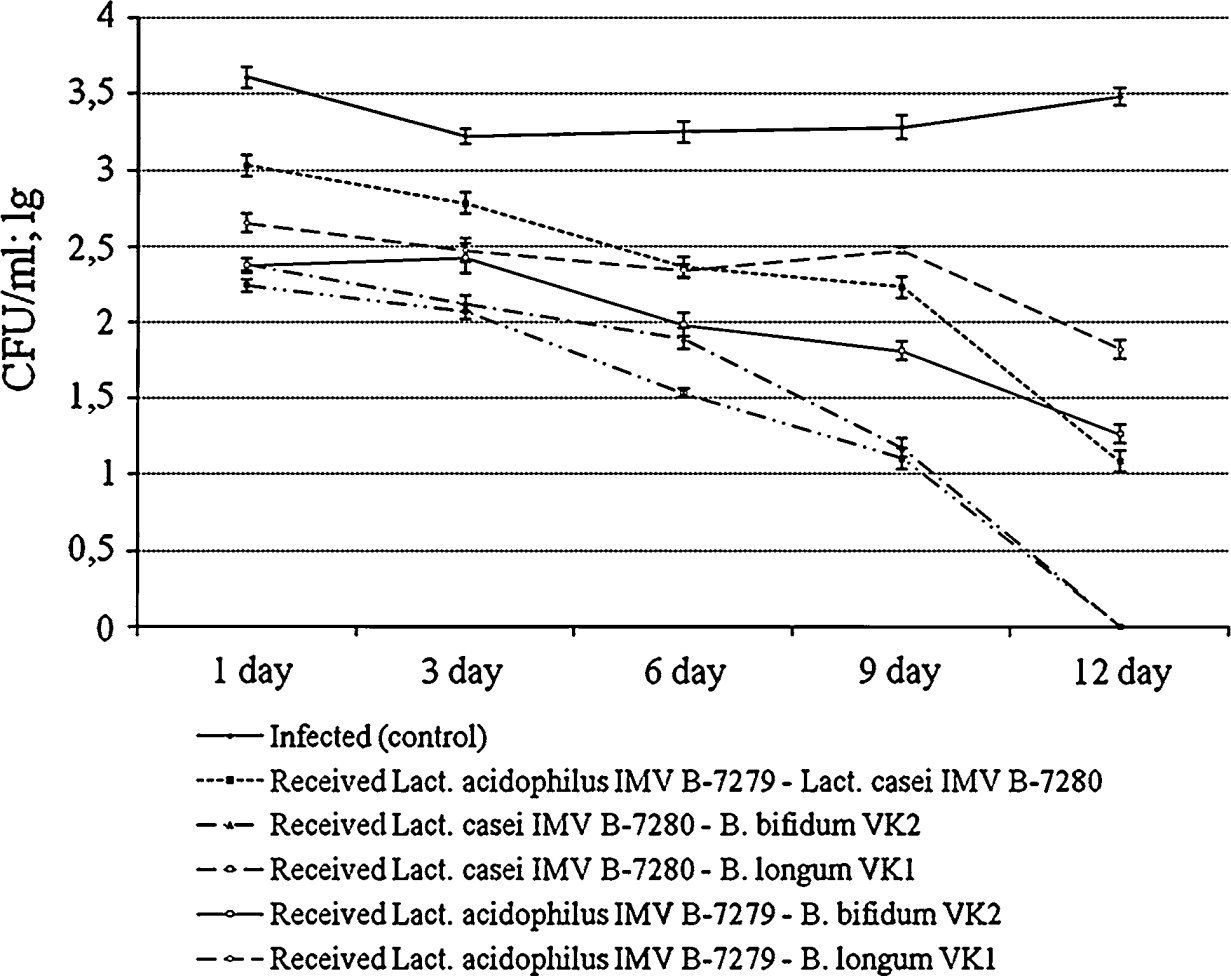
Number of colonies of *Staph. aureus* 8325-4, which was sowed from the vagina of the infected mice after receiving intravaginal injection of compositions of two probiotic strains of lactobacilli and bifidobacteria, each of them separately

The use of various compositions of probiotic cultures was also accompanied by a significant acceleration of the process of elimination of staphylococcus from the vagina. Comparing the anti-staphylococcal action of the compositions of two probiotic cultures (Fig. 2) in vivo, the combination of *Lact. casei* IMV B-7280 and *B. longum* VK1 (*P* < 0.05) had the most effective action within the entire period of observation. A rather high anti-staphylococcal effect was demonstrated by the *Lact. casei* IMV B-7280—*B. bifidum* VK2 composition (on the third, sixth, ninth, and twelfth days). After injection of *Lact. casei* IMV B-7280—*B. longum* VK1 or *Lact. casei* IMV B-7280—*B. bifidum* VK2 compositions into the infected mice, staphylococcus was completely eliminated from the vagina by the twelfth day. Compared with these two compositions of probiotic bacteria, the *Lact. acidophilus* IMV B-7279—*B*. *bifidum* VK2 composition proved to be less effective (*P* < 0.05). The *Lact.* *acidophilus* IMV B-7279—*B. longum* VK1 (on the sixth, ninth, and twelfth days; *P* < 0.05) and *Lact. casei* IMV B-7280—*Lact.* *acidophilus* IMV B-7279 (on the first, third, and sixth days; *P* < 0.05) compositions had even lower anti-staphylococcal activity compared with other compositions of the two strains. After injecting the *Lact. acidophilus* IMV B-7279—*B*. *bifidum* VK2, *Lact.* *acidophilus* IMV B-7279—*B. longum* VK1, or *Lact. casei* IMV B-7280—*Lact.* *acidophilus* IMV B-7279 compositions separately into the mice, staphylococcus was recovered from the vagina on the twelfth day, but at a much smaller amount than that from the infected mice who did not receive compositions of probiotic cultures. It is worth noting that, contrary to other compositions of the two strains, it was the *Lact. casei* IMV B-7280—*B. longum* VK1 composition that was a more active antagonist of staphylococcus during the entire period of observation than *Lact. casei* IMV B-7280 or *B. longum* VK1 separately.

**Fig. 3:**
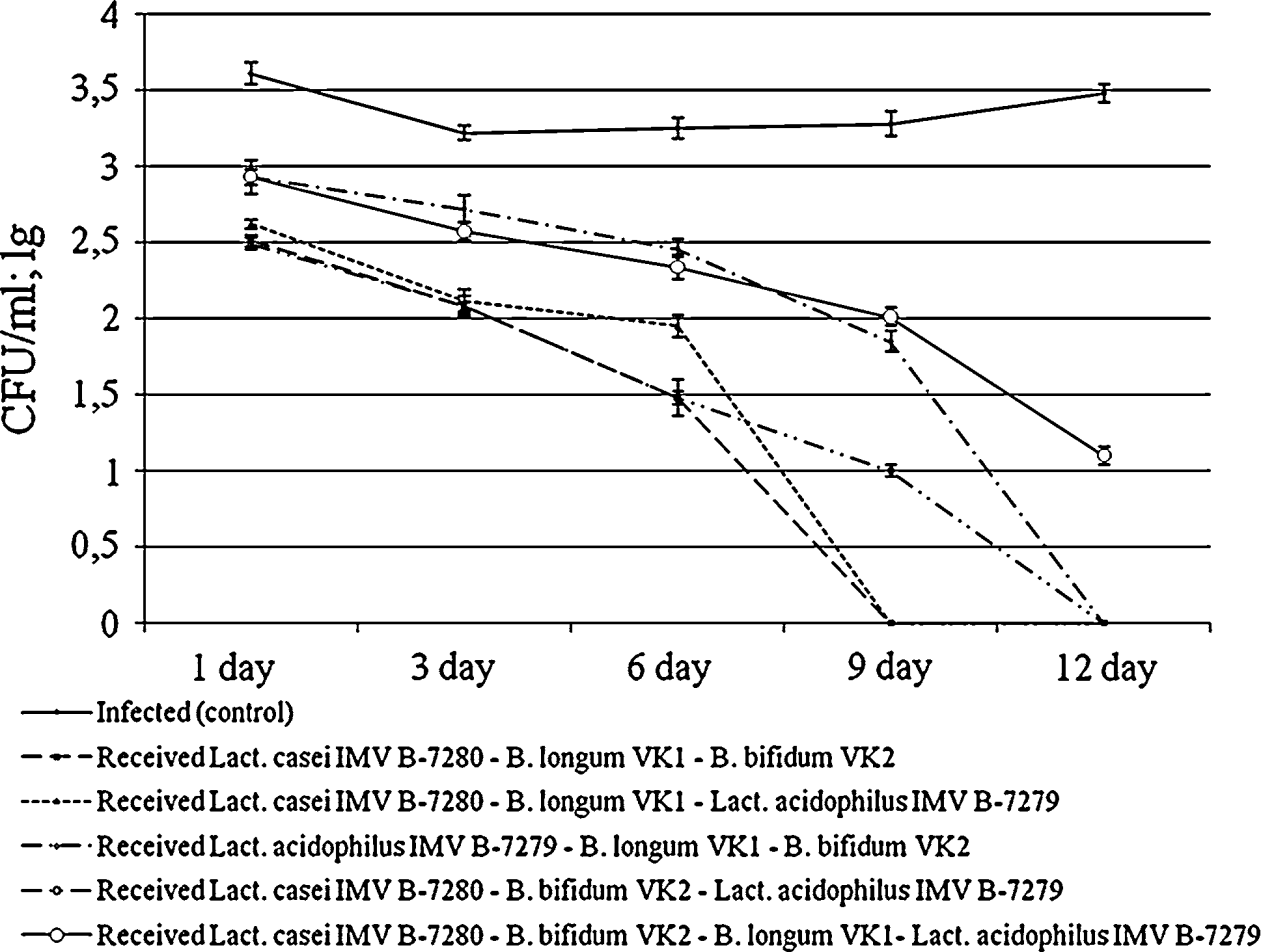
Number of colonies of *Staph. aureus* 8325-4, which was sowed from the vagina of the infected mice after receiving intravaginal injection of compositions of three or four probiotic strains of lactobacilli and bifidobacteria, each of them separately

As shown in Fig. 3, among the compositions of the three strains of probiotic cultures, *Lact. casei* IMV B-7280—*B. longum* VK1—*B. bifidum* VK2 and *Lact. casei* IMV B-7280—*B. bifidum* VK2—*Lact. acidophilus* IMV B-7279 had the best anti-staphylococcal action on the first, third, and sixth days, compared with other compositions of three and four strains of bacteria (*P* < 0.05). The efficiency of the *Lact. casei* IMV B-7280—*B. longum* VK1—*Lact. acidophilus* IMV B-7279 composition appeared to be the same on the third day as that of the two previously mentioned triple compositions, but the activity was less on the first and sixth days (*P* < 0.05). The lowest anti-staphylococcal activity on the first, third, and sixth days was demonstrated by the *Lact. acidophilus*—*B. longum* VK1—*B. bifidum* VK2 (*P* < 0.05) and *Lact. casei* IMV B-7280—*B. bifidum* VK2—*B. longum* VK1—*Lact. acidophilus* IMV B-7279 (*P* < 0.05) compositions, compared to other compositions of three strains. On the ninth and twelfth days, *Staph. aureus* 8325-4 was not recovered from the vagina of the infected mice who received the *Lact. casei* IMV B-7280—*B. longum* VK1—*B. bifidum* VK2 or *Lact. casei* IMV B-7280—*B. longum* VK1—*Lact. acidophilus* IMV B-7279 compositions. After injection of the *Lact. acidophilus*—*B. longum* VK1—*B. bifidum* VK2, *Lact. casei* IMV B-7280—*B. bifidum* VK2—*Lact. acidophilus* IMV B-7279, or *Lact. casei* IMV B-7280—*B. bifidum* VK2—*B. longum* VK1—*Lact. acidophilus* IMV B-7279 compositions to the mice, *Staph. aureus* 8325-4 was recovered from the vagina of the infected mice on the ninth day. From the vagina of the mice who received the composition of the four strains (*Lact. casei* IMV B-7280—*B. bifidum* VK2—*B. longum* VK1—*Lact. acidophilus* IMV B-7279), *Staph. aureus* 8325-4 was also recovered on the twelfth day.

Analyzing the data obtained, it is possible to conclude that the *Lact. casei* IMV B-7280—*B. longum* VK1—*B. bifidum* VK2 composition appeared to be the biggest antagonist of *Staph. aureus* 8325-4. The anti-staphylococcal activity of the *Lact. casei* IMV B-7280—*B. longum* VK1—*B. bifidum* VK2 composition on the first day after injection into the mice was the same as after injecting these monocultures separately. However, on the third, sixth, and ninth days, the anti-staphylococcal activity of this composition appeared to be better than that of the monocultures injected separately. On the twelfth day, *Staph. aureus* 8325-4 was still recovered out from the vagina of infected mice who received *B. longum* VK1 separately. On the first day, the efficiency of the *Lact. casei* IMV B-7280—*B. longum* VK1—*B. bifidum* VK2 composition was lower than that of the *Lact. casei* IMV B-7280—*B. longum* VK1 and *Lact. casei* IMV B-7280—*B. bifidum* VK2 compositions, but on the third and sixth days it was the same as that of the compositions of two strains. At the same time, on the ninth day, staphylococcus was fully eliminated from the vagina of the mice that received the *Lact. casei* IMV B-7280—*B. longum* VK1—*B. bifidum* VK2 composition, but was still recovered after the injection of the *Lact. casei* IMV B-7280—*B. longum* VK1 or *Lact. casei* IMV B-7280—*B. bifidum* VK2 compositions into the mice. Thus, comparing the strains separately and in various compositions with each other, it was determined that the *Lact. casei* IMV B-7280—*B. longum* VK1—*B. bifidum* VK2 composition is the most promising combination for the creation of a probiotic drug with anti-staphylococcal activity.

### Immune-Modulating Effect of the Lactobacilli and Bifidobacteria Strains

Cellular and humoral immunity are activated in the course of development of the immune response of an organism against staphylococci, because in the pathogenesis of diseases caused by staphylococcus, a certain role is played by both bacterial cells and their exotoxins. Therefore, the study of the number of CD3+ T-lymphocytes and their specific subpopulations (CD4+ T-helper cells, CD8+ T-suppressors), and CD19+ B-lymphocytes in the spleen of mice partly allows us to assess whether the strains of lactobacilli and bifidobacteria influenced the development of a systemic immune response in cases of intravaginal staphylococcosis. It was shown that the number of CD3+, CD4+, CD8+, and CD19+ cells, as well as the CD4/CD8 index, changed in cases of intravaginal staphylococcal infection (Table 2). After infection of the mice with staphylococcus on the third day, a reduction in the number of CD3+ and CD4+ cells in the spleen was observed, while the level of CD8+ cells was normal, compared to the control level (intact mice), demonstrating the development of a systemic immune response to staphylococcus. By reducing the number of CD4+ cells on the third day, a decrease in the CD4/CD8 index was observed in the infected mice. At the other times of observation (first, sixth, and ninth days), the number of these cells and the CD4/CD8 index were preserved at the control level. The number of CD19+ cells in the spleens of the infected mice did not change on the first and third days, increased more than twice (*P* < 0.05) on the sixth day, and decreased to the control level on the ninth day.

**Table 2:** Numbers of T-, B-lymphocytes in the spleens of mice receiving injections of *Lact. casei* IMV B-7280, *Lact. acidophilus* IMV B-7279, *B. longum* VK1, or *B. bifidum* VK2 separately

Groups of mice/time of observation, day	Relative number of cells, %				CD4/CD8, nominal units
	CD3+	CD4+	CD8+	CD19+	
Intact	61.9 ± 2.5	38.6 ± 1.3	26.3 ± 4.9	7.1 ± 0.5	1.5 ± 0.1
Infected					
First day	63.8 ± 1.3	38.6 ± 0.4	22.8 ± 0.1	6.7 ± 0.1	1.7 ± 0.5
Third day	53.3 ± 0.7*	29.5 ± 0.5*	27.1 ± 0.5	10.5 ± 1.7	1.0 ± 0.1*
Sixth day	56.3 ± 3.2	37.5 ± 0.7	23.6 ± 2.3	15.3 ± 1.6*	1.6 ± 0.3
Ninth day	56.6 ± 8.5	36.9 ± 2.5	24.1 ± 2.2	9.6 ± 2.7	1.5 ± 0.2
Received *Lact. acidophilus* IMV B-7279					
First day	53.3 ± 2.5*	34,0 ± 2.1	22.1 ± 5.3	11.3 ± 1.0*	1.6 ± 0.7
Third day	52.3 ± 1.3*	32.1 ± 1.2*	21.1 ± 1.9	10.4 ± 1.7	1.5 ± 0.1^•^
Sixth day	56.4 ± 4.1	35.0 ± 1.4	23.5 ± 0.7	10.1 ± 1.1	1.5 ± 0.4
Ninth day	60.5 ± 2.0	40.1 ± 0.1	26.3 ± 0.2	9.9 ± 2.2	1.5 ± 0.2
Received *Lact. casei* IMV B-7280					
First day	59.5 ± 3.8	35.0 ± 3.6	17.7 ± 4.5	8.7 ± 1.3	1.9 ± 0.4
Third day	58.0 ± 0.8^•^	38.3 ± 0.5^•^	25.7 ± 0.5	8.5 ± 1.9	1.5 ± 0.1^•^
Sixth day	59.8 ± 3.6	39.2 ± 3.7	23,9 ± 0,3	7.2 ± 0.7	1.6 ± 0.5
Ninth day	59.3 ± 2.9	38.2 ± 3.3	21.1 ± 4.0	8.3 ± 2.2	1.8 ± 0.4
Received *B. longum* VK1					
First day	55.0 ± 1.9	33.0 ± 5.9	21.4 ± 3.0	12.1 ± 0,9*	1.5 ± 0.2
Third day	56.8 ± 2.9	38.9 ± 1.1^•^	17.0 ± 4.1	5.0 ± 0.9	2.3 ± 0.2*^•^
Sixth day	55.9 ± 3.7	32.8 ± 3.0	23.4 ±3.5	6.7 ± 1.0	1.4 ± 0.4
Ninth day	62.9 ± 5.9	41.3 ± 3.8	26.0 ± 1.0	6.0 ± 2.9	1.6 ± 0.2
Received *B. bifidum* VK2					
First day	61.9 ± 3.9	40.5 ± 1.5	21.0 ± 2.2	11.2 ± 1.7*	1.9 ± 0.1
Third day	54.1 ± 1.8*	32.6 ± 0.9*	22.3 ± 1.9	6.8 ± 3.9	1.5 ± 0.1^•^
Sixth day	58.8 ± 2.2	40.3 ± 5.0	21.5 ± 4.0	10.4 ± 1.5	1.9 ± 0.4
Ninth day	55.0 ± 6.1	33.2 ± 4.1	21.4 ± 3.9	13.3 ± 1.1*	1.6 ± 0.3

Significant differences with the control is represented by * (*P* < 0.05) while differences with the indicators of the infected mice who did not receive probiotic strains or their compositions are represented by ^•^ (*P* < 0.05)

After the injection of some individual probiotic cultures and some of their compositions into the infected mice, an increase in the level of indicators controlling the number of CD3+ and/or CD4+ cells (third day) in the spleen was observed, while the level of CD8+ cells was normal, and an increase in the number of CD19+ cells was observed in different periods. It was found (Table 3) that the number of CD3+ and CD4+ cells on the third day in the spleens of infected mice increased under the influence of *Lact. casei* IMV B-7280 compared with the infected mice that did not receive the probiotic cultures. Following injection of *B. longum* VK1 into the infected mice, on the third day, only a tendency to increase in the number of CD3+ cells and a probable increase in the number of CD4+ cells was observed. The number of CD3+ and CD4+ cells was smaller on the third day in the spleen of infected mice who received either *Lact.* *acidophilus* IMV B-7279 or *B. bifidum* VK2 separately than in the control. After the injection of *Lact.* *acidophilus* IMV B-7279 into the infected mice, the number of CD3+ cells on the first day appeared to be lower than in the control. However, it should be noted that after the injection of all these probiotic cultures into the infected mice, a separate increase in the CD4/CD8 index was detected on the third day compared with the infected mice who did not receive probiotic cultures. This may be due to an increase in the number of CD4+ cells under the influence of *Lact. casei* IMV B-7280 or *B. longum* VK1, or due to the reduction in the number of CD8+ cells under the influence of *Lact.* *acidophilus* IMV B-7279 or *B. bifidum* VK2. At other times of observation following the injection of these probiotic cultures separately into the infected mice, the number of CD3+, CD4+, and CD8+ cells and the CD4/CD8 index were preserved on the level of control. It was shown that in the spleen of infected mice, the number of CD19+ cells also increased on the first day after the injection of *Lact.* *acidophilus* IMV B-7279 or *B. longum* VK1 separately, and on the first and ninth days after the injection of *B. bifidum* VK2. The number of CD19+ cells did not change in the spleens of infected mice that received *Lact. casei* IMV B-7280.

**Table 3:** Numbers of T-, B-lymphocytes in the spleens of mice who received compositions of two strains of *Lact. casei* IMV B-7280, *Lact. acidophilus* IMV B-7279, *B. longum* VK1, or *B. bifidum* VK2

Groups of mice/time of observation, day	Relative number of cells, %				CD4/CD8, nominal units
	CD3+	CD4+	CD8+	CD19+	
Intact	61.9 ± 2.5	38.6 ± 1.3	26.3 ± 4.9	7.1 ± 0.5	1.5 ± 0.1
Infected					
First day	63.8 ± 1.3	38.6 ± 0.4	22.8 ± 0.1	6.7 ± 0.1	1.7 ± 0.4
Third day	53.3 ± 0.7*	29.5 ± 0.5*	27.1 ± 0.5	10.5 ± 1.7	1.0 ± 0.1
Sixth day	56.3 ± 3.2	37.5 ± 0.7	23.6 ± 2.3	15.3 ± 1.6*	1.6 ± 0.5
Ninth day	56.6 ± 2.5	36.9 ±.,5	24.1 ± 2.2	9.6 ± 2.7	1.5 ± 0.2
Received *Lact. casei* IMV B-7280—*B. bifidum* VK2					
First day	61.9 ± 0.9	40.2 ± 4.5	21.7 ± 3.5	7.4 ± 0.5	1.9 ± 0.4
Third day	61.6 ± 1.0^•^	38.2 ± 1.1^•^	23.0 ± 2.4	9.1 ± 1.5	1.7 ± 0.1^•^
Sixth day	55.1 ± 9.5	34.0 ± 2.9	16.9 ± 5.1	8.0 ± 0.5	2.0 ± 0.1^•^*
Ninth day	55.0 ± 5.1	38.2 ± 3.8	16.4 ± 3.4	6.0 ± 3.4	2.3 ± 0.2^•^*
Received *Lact. casei* IMV B-7280—*B. longum* VK1					
First day	62.2 ± 3.0	32.4 ± 2.6	19.2 ± 2.9	10.1 ± 5.1	1.7 ± 0.3
Third day	59.3 ± 1.5^•^	36.7 ± 1.0^•^	19.6 ± 6.1	12.0 ± 1.0*	1.9 ± 0.1^•^
Sixth day	57.0 ± 1.3	37.0 ± 2.8	24.6 ± 3.1	9.4 ± 2.1	1.5 ± 0.4
Ninth day	55.0 ± 2.9	37.1 ± 3.0	22.5 ± 7.5	12.4 ± 1.5*	1.6 ± 0.5
Received *Lact. acidophilus* IMV B-7279—*B. bifidum* VK2					
First day	55.1 ± 4.1	34.9 ± 4.2	21.6 ± 4.0	9.0 ± 4.1	1.6 ± 0.2
Third day	59.2 ± 0.5^•^	37.6 ± 3.1^•^	23.4 ± 3.1	10.1 ± 6.2	1.6 ± 0.1^•^
Sixth day	64.6 ± 2.5	41.9 ± 4.0	24.4 ± 4.2	14.6 ± 1.0*	1.7 ± 0.3
Ninth day	61.3 ± 2.0	38.0 ± 2.4	26.4 ± 3.2	10.6 ± 4.6	1.4 ± 0.1
Received *Lact.* *acidophilus* IMV B-7279—*B. longum* VK1					
First day	57.1 ± 4.1	39.0 ± 4.9	24.1 ± 5.1	13.7 ± 3.5*	1.6 ± 0.2
Third day	51.9 ± 1.5*	30.4 ± 1.2*	22.2 ± 3.1	6.3 ± 2.9	1.4 ± 0.1
Sixth day	56.5 ± 4.1	35.0 ± 3.5	20.8 ± 7.2	10.4 ± 2.2	1.6 ± 0.3
Ninth day	57.0 ± 3.0	36.3 ± 5.6	20.4 ± 4.1	9.1 ± 1.5	1.8 ± 0.5
Received *Lact. acidophilus* IMV B-7279—*Lact. casei* IMV B-7280					
First day	50.1 ± 1.5*	31.8 ± 2.1*	18.6 ± 5.1	9.9 ± 2.0	1.7 ± 0.1
Third day	53.0 ± 2.1*	31.0 ± 2.0*	18.0 ± 4.6	11.3 ± 2.1*	1.7 ± 0.1^•^
Sixth day	60.5 ± 3.5	35.7 ± 3.1	24.0 ± 3.5	7.5 ± 1.5	1.5 ± 0.3
Ninth day	61.3 ± 2.0	38.0 ± 2.7	26.4 ± 1.5	10.6 ± 2.0	1.4 ± 0.2

Significant differences with the control is represented by * (*P* < 0.05), while differences with the indicators of the infected mice who did not receive probiotic strains or their compositions are represented by ^•^ (*P* < 0.05)

An increase in the number of CD3+ and CD4+ cells to the control level was observed on the third day in the spleens of infected mice under the influence of the following compositions of two probiotic cultures (Table 3): *Lact. casei* IMV B-7280—*B. bifidum* VK2, *Lact. casei* IMV B-7280—*B. longum* VK1 or *Lact. acidophilus* IMV B-7279—*B. bifidum* VK2. Injection of the *Lact.* *acidophilus* IMV B-7279—*B. longum* VK1 or *Lact.* *acidophilus* IMV B-7279—*Lact. casei* IMV B-7280 composition separately into the infected mice on the third day did not cause the increase in the number of CD3+ and CD4+ cells; in the spleens of infected mice injected with the *Lact.* *acidophilus* IMV B-7279—*Lact. casei* IMV B-7280 composition, the number of these cells also appeared to be low on the first day compared with both the control and the infected mice that did not receive the composition of probiotic cultures. The CD4/CD8 index rose on the third day following the injection of the *Lact. casei* IMV B-7280—*B. bifidum* VK2 composition into the infected mice (due to an increase in the number of CD4+ cells) and also on the sixth and ninth days (due to the tendency to reduction in the number of CD8+ cells). After *Lact. casei* IMV B-7280—*B. longum* VK1 or *Lact. acidophilus* IMV B-7279—*B. bifidum* VK2 injection, the CD4/CD8 index rose only on the third day (due to increase in the number of CD4+ cells). Under the influence of the *Lact.* *acidophilus* IMV B-7279—*B. longum* VK1 composition, the tendency of the CD4/CD8 index to increase on the third day was noted. However, after the injection of the *Lact.* *acidophilus* IMV B-7279—*Lact. casei* IMV B-7280 composition into the infected mice, this indicator also increased on the third day (probably due to the tendency of the number of CD8+ cells to decrease). The number of CD19+ cells, compared with the indicators of control (intact mice), did not change under the influence of the *Lact. casei* IMV B-7280—*B. bifidum* VK2 composition, but did increase after the injection of the following compositions into the infected mice: *Lact. casei* IMV B-7280—*B. longum* VK1 (on the third and ninth days), *Lact.* *acidophilus* IMV B-7279—*B. bifidum* VK2 (on the sixth day), *Lact.* *acidophilus* IMV B-7279—*B. longum* VK1 (on the first day), *Lact.* *acidophilus* IMV B-7279—*Lact. casei* IMV B-7280 (on the third day) (Table 3). The number of CD19+, CD3+, CD4+, and CD8+ cells, and the CD4/CD8 index were preserved at the level of control after the injection of the compositions of two strains of probiotic bacteria into the infected mice at other times of observation.

The number of CD3+ and CD4+ cells in the spleens of infected mice increased up to the level of the control on the third day under the influence of the following compositions of three or four strains (Table 4): *Lact. casei* IMV B-7280—*B. longum* VK1—*Lact. acidophilus* IMV B-7279, *Lact. acidophilus* IMV B-7279—*B. longum* VK1—*B. bifidum* VK2 or *Lact. casei* IMV B-7280—*B. bifidum* VK2—*B. longum* VK1—*Lact. acidophilus* IMV B-7279. After the injection of the *Lact. casei* IMV B-7280—*B. longum* VK1—*B. bifidum* VK2 or *Lact. casei* IMV B-7280—*B. bifidum* VK2—*Lact. acidophilus* IMV B-7279 compositions, the number of CD4+ cells rose up on the third day compared with the indicators for the infected mice who did not receive these compositions, although the number of CD3+ cells remained low compared to the control indicators. On the third day, an increase in the CD4/CD8 index was observed due to an increase in the number of CD4+ cells after the injection of the following compositions separately into the infected mice compared with the infected mice who did not receive the probiotic bacteria: *Lact. casei* IMV B-7280—*B. longum* VK1—*B. bifidum* VK2, *Lact. casei* IMV B-7280—*B. longum* VK1—*Lact. acidophilus* IMV B-7279, *Lact. casei* IMV B-7280—*B. bifidum* VK2—*Lact acidophilus* IMV B-7279 or *Lact. casei* IMV B-7280—*B. bifidum* VK2—*B. longum* VK1—*Lact. acidophilus* IMV B-7279. After the injection of the *Lact. casei* IMV B-7280—*B. longum* VK1—*B. bifidum* VK2 composition into the infected mice, this indicator also increased on the first day. Only on the third day, a tendency of the CD4/CD8 index to increase was detected under the influence of the all studied groups, but for the *Lact. acidophilus*—*B. longum* VK1—*B. bifidum* VK2 composition, this indicator rose on the first day. It was shown that the number of CD19+ cells (Table 4) increased after the injection of the following compositions of strains into the infected mice: *Lact. casei* IMV B-7280—*B. longum* VK1—*B. bifidum* VK2 (on the ninth day), *Lact. casei* IMV B-7280—*B. bifidum* VK2—*Lact. acidophilus* IMV B-7279 (on the third day), *Lact. casei* IMV B-7280—*B. longum* VK1—*Lact. acidophilus* IMV B-7279 (on the first, third, and sixth days) or *Lact. casei* IMV B-7280—*B. bifidum* VK2—*B. longum* VK1—*Lact. acidophilus* IMV B-7279 (on the first, third, and sixth days) (Table 4). After the injection of the *Lact. acidophilus* IMV B-7279—*B. longum* VK1—*B. bifidum* VK2 composition into the infected mice, the number of CD19+ cells did not change compared to the control indicators. At other times of observation, after the injection of compositions of three or four probiotic bacteria into the infected mice, the number of CD19+, CD3+, CD4+, and CD8+ cells, and the CD4/CD8 index were preserved at the level of the control indicators.

**Table 4:** Numbers of T-, B-lymphocytes in the spleens of mice who received compositions of three or four strains of *Lact. casei* IMV B-7280, *Lact. acidophilus* IMV B-7279, *B. longum* VK1, or *B. bifidum* VK2

Groups of mice/time of observation, day	Relative number of cells, %				CD4/CD8, arbitrary units
	CD3+	CD4+	CD8+	CD19+	
Intact	61.9 ± 2.5	38.6 ± 1.3	26.3 ± 4.9	7.1 ± 0.5	1.5 ± 0.1
Infected					
First day	63.8 ± 1.3	38.6 ± 0,4	22.8 ± 0,1	6.7 ± 0.1	1.7 ± 0.1
Third day	53.3 ± 0,7*	29.5 ± 0.5*	27.1 ± 0,5	10.5 ± 1.7	1.0 ± 0.1
Sixth day	56.3 ± 3.2	37.5 ± 0.7	23.6 ± 2,3	15.3 ± 1.6*	1.6 ± 0.1
Ninth day	56.6 ± 8.5	36.9 ± 2.5	24.1 ± 2,2	9.6 ± 2.7	1.5 ± 0.1
Received *Lact. casei* IMV B-7280—*B. longum* VK1—*B. bifidum* VK2					
First day	51.2 ± 2.9	38.9 ± 1.7	19.6 ± 4.7	8.7 ± 0.9	2.0 ± 0.1^*^
Third day	54.1 ± 1.3*	36.0 ± 0.5^•^	21.5 ± 1.1	6.1 ± 1.7	1.7 ± 0.1^•^
Sixth day	57.0 ± 2.7	40.8 ± 2.7	28.1 ± 2.0	10.3 ± 0.9	1.5 ± 0.1
Ninth day	56.0 ± 3.1	37.8 ± 1.0	28.6 ± 3.2	11.7 ± 1.1*	1.4 ± 0.1
Received *Lact. casei* IMV B-7280—*B. longum* VK1—*Lact. acidophilus* IMV B-7279					
First day	55.1 ± 1.6	32.7 ± 2.1	23.1 ± 4.0	18.3 ± 1.7*	1.4 ± 0.1
Third day	58.0 ± 1.4^•^	40.2 ± 1.0^•^	26.4 ± 2.4	11.5 ± 0.9*	1.5 ± 0.1^•^
Sixth day	57.0 ± 3.9	34.0 ± 2.7	22.9 ± 1.7	15.2 ± 1.7*	1.5 ± 0.1
Ninth day	58.9 ± 2.1	37.6 ± 3.3	26.5 ± 2.0	9.0 ± 2.1	1.4 ± 0.1
Received *Lact. acidophilus* IMV B-7279—*B. longum* VK1—*B. bifidum* VK2					
First day	54.6 ± 3.2	38.3 ± 5.7	18.2 ± 3.1	6.3 ± 1.4	2.1 ± 0.1*
Third day	63.0 ± 0.7^•^	36.0 ± 1.1^•^	27.8 ± 4.0	10.0 ± 3.2	1.3 ± 0.1
Sixth day	55.0 ± 2.9	37.3 ± 3.4	23.4 ± 2.9	8.1 ± 2.7	1.6 ± 0.1
Ninth day	54.8 ± 3.1	35.5 ± 2.2	19.1 ± 3.7	8.2 ± 1.1	1.8 ± 0.1
Received *Lact. casei* IMV B-7280—*B. bifidum* VK2—*Lact. acidophilus* IMV B-7279					
First day	57.3 ± 3.2	38.9 ± 3.0	23.8 ± 2.8	8.1 ± 2.3	1.6 ± 0.1
Third day	53.0 ± 2.6*	35.4 ± 0.9^•^	18.8 ± 4.9	13.1 ± 1.1*	1.9 ± 0.1^•^
Sixth day	60.3 ± 1.3	40.1 ± 2.4	26.2 ± 5.0	8.9 ± 2.4	1.5 ± 0.1
Ninth day	61.1 ± 3.5	41.7 ± 1.8	24.1 ± 2.9	7.7 ± 2.0	1.7 ± 0.1
Received *Lact. casei* IMV B-7280—*B. bifidum* VK2—*B. longum* VK1—*Lact. acidophilus* IMV B-7279					
First day	55.5 ± 1.7	37.3 ± 4.0	24.4 ± 4.7	16.7 ± 3.8*	1.5 ± 0.1
Third day	59.4 ± 1.0^•^	41.6 ± 1.3^•^	24.3 ± 1.8	11.7 ± 0.9*	1.7 ± 0.1^•^
Sixth day	56.1 ± 3.5	35.0 ± 2.7	23.6 ± 2.5	18.3 ± 1.1*	1.5 ± 0.1
Ninth day	58.9 ± 2.9	39.3 ± 3.1	27.2 ± 2.0	6.1 ± 0.9	1.4 ± 0.1

Significant differences with the control is represented by * (*P* < 0.05), while differences with the indicators of the infected mice who did not receive probiotic strains or their compositions are represented by ^•^ (*P* < 0.05)

Thus, *Lact. casei* IMD-7280 alone, as well as most compositions of strains of lactobacilli and bifidobacteria (except for the *Lact.* *acidophilus* IMV B-7279—*B. longum* VK1 and *Lact. acidophilus* IMV B-7279—*Lact. casei* IMV B-7280 compositions) which were studied by us, caused a normalization of cellular immunity indicators: On the third day, the number of CD3+ and/or CD4+ cells increased in the spleens of the infected mice compared with those from infected mice that did not receive probiotic cultures or their compositions. At the same time, after the injection of *Lact.* *acidophilus* IMV B-7279 or *B. bifidum* VK2 separately, or the composition of *Lact.* *acidophilus* IMV B-7279—*B. longum* VK1 and *Lact.* *acidophilus* IMV B-7279—*Lact. casei* IMV B-7280, into the infected mice, normalization of the number of CD3+ and CD4+ cells was not observed. However, these strains of bacteria and their varied compositions did increase the number of CD19+ cells in various periods of observation. An increase in the number of CD19+ cells in the spleens of the infected mice was also detected after the injection of other strains of bacteria and compositions, except for *Lact. casei* IMV B-7280 and the *Lact. casei* IMV B-7280—*B. bifidum* VK2 and *Lact. acidophilus* IMV B-7279—*B. longum* VK1—*B. bifidum* VK2 compositions. At the same time, *Lact. casei* IMV B-7280 or the *Lact. casei* IMV B-7280—*B. bifidum* VK2 or *Lact. acidophilus* IMV B-7279—*B. longum* VK1—*B. bifidum* VK2 compositions did not influence the number of CD19+ cells in the spleens of the infected mice compared to the control indicators (intact mice).

## Discussion

To determine the possibility of creating probiotic compositions based on strains of lactobacilli and bifidobacteria for treatment of patients with dysbiosis and uncomplicated infections of the urogenital tract, we performed a comprehensive study of the antibacterial and immune modulatory activities of the strains *Lact. casei* IMV B-7280, *Lact.* *acidophilus* IMV B-7279, *B. longum* VK1, *B. bifidum* VK2, and their various compositions. The model used was experimental intravaginal infection of mice caused by *Staph. aureus* [31], which is a frequent cause of dysbiosis and of development of uncomplicated infections of the human urogenital tract. It is considered that the use of probiotics based on lactobacilli and bifidobacteria in the treatment of such patients is an alternative therapy to antibiotics and chemotherapy [8]. Studies on the antagonistic interactions between lactic acid bacteria and *Staph. aureus* have been carried out in various laboratories throughout the world over the past several decades [2]. It has been repeatedly shown that many strains of lactobacilli and bifidobacteria taken from the intestines and vagina inhibit the growth of *Staph. aureus* in vitro. For example, *Lact. acidophilus* EP317/402 [28], *Lact. acidophilus* CL1285(^®^) and *Lact. casei* LBC80R [9]; *Lact. plantarum* 8P-A3, *Lact. casei* DN-114001, *Lact. reuteri* [4], *B.* *longum* Z4, *B. bifidum* Г1 [11], and some other strains of *Bifidobacterium* [12] had antagonistic activity in vitro in relation to *Staph. aureus*. On the mouse model of intravaginal staphylococcosis, *Lact. paracasei* CRL 1289 prevented vaginal colonization of a uropathogenic strain of *Staph. aureus*, which was confirmed by the reduction in cell numbers, the normalization of inflammation, and the cytomorphological structure of the vaginal mucous membrane [35]. *Lact. rhamnosus* GR-1, *Lact. fermentum* RC-14 and *Lact. crispatus* CTV-05 also demonstrated their effectiveness against agents of infectious diseases of the urogenital tract both in vitro and on animal models [6, 17, 21, 24, 25]. The efficacy and safety of *Lact. rhamnosus* GR-1 and *Lact. fermentum* RC-14 as treatments were proven in several clinical studies of intravaginal application [23] and their consumption in milk [22]. At the same time, the search for other strains of lactic acid bacteria that are highly effective against staphylococci and could be used to create probiotic drugs remains the issue of the day. All of the probiotic bacterial strains tested in monoculture showed significant anti-staphylococcal activity on in vivo models of experimental intravaginal infection of mice induced by *Staph. aureus* 8325-4. However, their anti-staphylococcal action in vitro and in vivo was different. The in vitro antagonistic activity in relation to strains of *Staph. aureus* 8325-4 can be assessed as follows: *Lact.* *acidophilus* IMV B-7279 > *B. bifidum* VK2 > *B. longum* VK1/*Lact. casei* IMV B-7280. Thus, in vivo *B. bifidum* VK2 or *Lact. casei* IMV B-7280 showed the highest individual antagonistic activity against *Staph. aureus* 8325-4. This may be explained by the fact that probiotic bacteria can use different mechanisms of anti-staphylococcal action in vitro and in vivo. Primarily, this is due to their competitiveness with the pathogen [34, 35] and with different levels of adhesion to epithelial cells. Indeed, *Lact. casei* IMV B-7280, *B. bifidum* VK2, and *B. longum* VK1 have shown higher levels of adhesion to epithelial cells than *Lact.* *acidophilus* IMV B-7279 (unpublished data). The important anti-staphylococcal mechanisms of action of lactobacilli and bifidobacteria in vivo are the production of lactic acid, hydrogen peroxide, bacteriocins-like compounds (antimicrobial peptides), and other biologically active substances [1, 16].We have not studied these in relation to *Lact. casei* IMV B-7280, *Lact.* *acidophilus* IMV B-7279, *B. longum* VK1, and *B. bifidum* VK2, and they will be the subject of future research.

Earlier, we demonstrated [29] that *Lact. casei* IMV B-7280, *Lact.* *acidophilus* IMV B-7279, *B. longum* VK1 and *B. bifidum* VK2 are not antagonists of each other. This allowed us to compose them in various compositions of two, three, or four probiotic cultures. Our data indicate that the studied probiotic cultures and their compositions have an immunomodulatory effect in the mice infected with *Staphylococcus*. We studied changes in the numbers of T- and B-lymphocytes, and certain subpopulations of T-lymphocytes in the spleen of the mice infected with *Staph. aureus* 8325-4. Experimental models and studies on patients have shown that the immunomodulatory mechanism of action of many probiotic strains of lactobacilli and bifidobacteria involves activation of cellular and humoral immunity: the numbers of T- and B-lymphocytes increases, as does their proliferative activity and production of a series of imunoregulatory cytokines [3, 10, 13, 32, 33].

As we previously established on the model of intact mice, *Lact. casei* IMV B-7280, *Lact.* *acidophilus* IMV B-7279, *B. longum* VK1, and *B. bifidum* VK2 intensified the production of endogenous interferon and activated macrophages [27]. In this study, we showed that under the influence of some strains, particularly *Lact. casei* IMV B-7280 or *B. longum* VK1 alone, or several of the compositions of multiple probiotic cultures, the number of CD3+ and/or CD4+ cells in the spleen of the infected mice increased to the level of control on the third day, compared with the infected mice who did not receive probiotic cultures or their compositions. After injection of the *Lact. casei* IMV B-7280—*B. longum* VK1—*B. bifidum* VK2 composition (which demonstrated the best anti-staphylococcal effect in vivo) into the mice infected with staphylococcus, an increase in the number of CD4+ cells was observed in the spleen on the third day, while the number of CD3+ cells was the same as in the infected mice who did not receive probiotic cultures or their compositions. Moreover, the number of CD19+ cells increased on the ninth day in comparison with the control indicators (intact mice). Combining *Lact. casei* IMV B-7280 and *B. longum* VK1 in one composition is successful due to normalization of the number of CD3+ and CD4+ cells observed in the spleens of the infected mice. In terms of their individual immune modulatory activities, the first strain induces the “late” interferon, while the second strain induces the “early” interferon, as we previously showed [18] on the model of intact mice. Thus, in our studies, there was a correlation between the ability of strains of lactobacilli and bifidobacteria or their compositions to inhibit the growth of *Staph. aureus* in vivo and their various immune modulatory properties. However, it should be noted that the immunomodulatory action of *Lact. casei* IMV B-7280 and *B. longum* VK1 separately, as well as some compositions of other strains, was better than that of the *Lact. casei* IMV B-7280—*B. longum* VK1—*B. bifidum* VK2 composition having the highest anti-staphylococcal action in vivo. The probiotic strains studied by us, individually and in various compositions, may use different mechanisms of influence on the development of the immune response.

Thus, *Lact. casei* IMV B-7280, *Lact. acidophilus* IMV B-7279, *B. longum* VK1, or *B. bifidum* VK2 can be used for creating probiotic drugs effective against *Staph. aureus* and having immunomodulatory effect. *Lact. casei* IMV B-7280—*B. longum* VK1—*B. bifidum* VK2 appeared to be the most promising composition. Before we can create a commercial probiotic drug on the basis of these strains of probiotic cultures for intravaginal use, further research must be conducted. We plan to determine the strains’ influence on the growth of opportunistic flora, especially fungi of the *Candida* genus, as well as on the balance of the production of pro- and anti-inflammatory cytokines, namely the Th1- and Th2-types of cytokines. Our results confirm the validity of the requirements of the European regulatory legislation in the field of probiotics regarding the need for comprehensive studies of biological activity of both separate cultures and their combinations, which would allow for the creation of effective probiotic drugs based on monocultures of lactobacilli and/or bifidobacteria or their various combinations.
